# Volatile Profiling of Strawberry Fruits Cultivated in a Soilless System to Investigate Cultivar-Dependent Chemical Descriptors

**DOI:** 10.3390/foods9060768

**Published:** 2020-06-11

**Authors:** Raúl González-Domínguez, Ana Sayago, Ikram Akhatou, Ángeles Fernández-Recamales

**Affiliations:** 1 AgriFood Laboratory, Faculty of Experimental Sciences, University of Huelva, 21007 Huelva, Spain; ana.sayago@dqcm.uhu.es (A.S.); ikram.akhatou@alu.uhu.es (I.A.); recamale@dqcm.uhu.es (Á.F.-R.); 2 International Campus of Excellence CeiA3, University of Huelva, 21007 Huelva, Spain

**Keywords:** strawberry, volatile profile, variety, soilless system

## Abstract

Volatile compounds are essential for food organoleptic characteristics and of great utility for the food industry as potential markers for authenticity purposes (e.g., variety, geographical origin, adulteration). The aim of this study was to determine the characteristic volatile compounds of strawberry samples grown in a soilless system by using headspace solid phase micro-extraction coupled with gas chromatography and to investigate the influence of cultivar (Festival, Candonga, Camarosa) on this volatile profile. We observed that Festival and, to a lesser extent, Candonga varieties were characterized by the richest aroma-related profiles, including higher levels of esters, furanones and terpenes. In particular, methyl butyrate, hexyl hexanoate, linalool, geraniol and furaneol were the most abundant aromatic compounds detected in the three varieties of strawberries. Complementarily, the application of pattern recognition chemometric approaches, including principal component analysis and linear discriminant analysis, demonstrated that concentrations of specific volatiles can be employed as chemical descriptors to discriminate between strawberry cultivars. Accordingly, geraniol and hexyl hexanoate were found to be the most significant volatiles for the discrimination of strawberry varieties.

## 1. Introduction

The typical fragrance of strawberry (*Fragaria* × *ananassa* Duch.) results from a balance between different volatile classes, comprising esters, aldehydes, ketones, alcohols, terpenes, furanones, and sulfides [1,2,3,4,5]. Among them, methyl and ethyl esters of butanoic and hexanoic acids, two furanones (furaneol and mesifuran) and methyl anthranilate have been described as the main contributors to the strawberry-like aroma [6,7,8]. The volatile content in foods is low compared with other nutrients and components, representing less than 0.01% of the fruit fresh weight in strawberry [6], but it is highly influenced by multiple factors, such as the cultivar, climate, production region, sampling time, the degree of ripeness, and post-harvest environment, among others [9,10,11,12,13]. In this vein, several authors have previously reported that some volatiles are highly cultivar-specific (e.g., ethyl butanoate, methyl anthranilate, furaneol, mesifuran), whereas others were found to be general strawberry aroma compounds (e.g., ethyl hexanoate) [4,14]. However, although numerous studies have been performed over the last years to investigate the impact of genotypic and agronomic factors on the chemical composition of strawberry, there is still scarce information about the volatile content of strawberries grown in soilless systems. The soilless culture has experienced strong development in southern regions of Spain, France and Italy due to the phase out of methyl bromide, a broad-spectrum soil fumigant traditionally employed for strawberry production [15]. In previous studies, the nutritional characteristics of strawberries grown in soilless systems have been compared with those cultivated in soil crops [16], and differences in color and phenolic content among five strawberry varieties cultivated in two soilless substrates have also been evaluated [17,18]. More recently, great efforts have been made to characterize the chemical composition of soilless-grown strawberry and to elucidate the effect of cultivar and agronomic practices by applying complementary analytical approaches, such as metabolomics [19], polyphenolic analysis [20], and the multi-targeted profiling of different nutrients and food components (e.g., sugars, organic acids, minerals) [21,22,23]. In this context, the study of the volatile fraction from strawberry cultivars grown in soilless systems could be of great interest for the food industry to select optimal breeding and cultivation strategies aimed to produce higher quality fruits with an enhanced aroma. Furthermore, these volatile compounds might serve as markers for detecting adulteration [24] and to predict the shelf life of derived food products and establish the best commercial storage conditions to preserve their quality [25].

Considering this lack of information about the chemical composition of strawberries grown in soilless systems and the effect of cultivar, the aim of this work was to investigate the characteristic volatile profile of strawberries cultivated using this non-conventional crop system. For this purpose, an analytical method based on headspace solid phase micro-extraction (HS-SPME) and gas chromatography (GC) was applied to investigate the effect of cultivar (Festival, Candonga, Camarosa) on these aromatic compounds. Then, two complementary chemometrics approaches were employed, namely principal component analysis and linear discriminant analysis.

## 2. Materials and Methods

### 2.1. Samples

The strawberry fruits of three varieties (Camarosa, Candonga and Festival) were grown in Huelva (southwest Spain; latitude 37° 14′ N, longitude 6° 53′ W, altitude 23 m) in a soilless system, as detailed elsewhere [22]. These cultivars were selected with the aim of investigating the impact of soilless growing on the volatile composition of strawberries with a different sensitivity to environmental conditions, from very sensitive varieties (e.g., Festival) to more resistant ones (e.g., Camarosa). Fruits were harvested at commercial ripeness (>75% of the surface showing red color), washed and sepals dissected. Then, the berries (100 g) were gently homogenized with 100 mL of a 20% NaCl solution (*w*/*v*) by using a kitchen mixer, snap-frozen in liquid nitrogen, and stored at −20 °C until analysis. For each cultivar, five independent homogenized samples were prepared by pooling 10 individual fruits from different plants.

### 2.2. Reagents and Chemicals

Absolute ethanol (>99.5%) and sodium chloride (99.5%) were purchased from Scharlab (Barcelona, Spain). Ultrapure water (18 MΩ cm^−1^) was obtained from a Milli-Q system (Millipore, Milford, MA, USA). The volatile standards, with purity above 99%, were supplied by Chemservice (West Chester, PA, USA) and Aldrich (Gillingham, UK): methyl butanoate, ethyl butanoate, methyl hexanoate, ethyl hexanoate, hexyl hexanoate, cis-3-hexenyl acetate, trans-2-hexenyl acetate, hexanal, trans-2-hexen-1-al, benzaldehyde, 1-hexanol, trans-2-hexen-1-ol, benzyl alcohol, linalool, geraniol, cis-nerolidol, nerol, mesifurane, furaneol, 2-methylbutanoic acid, 3-methylbutanoic acid, hexanoic acid, γ-nonalactone, and Δ-decalactone. 2-octanol (internal standard, IS) was obtained from Fluka (Madrid, Spain). Individual stock solutions at 1000 mg L^−1^ for each compound and IS were prepared in ethanol and stored at −20 °C.

### 2.3. Headspace Solid-Phase Micro-Extraction Procedure

A manual fiber holder and 65 μm polydimethylsiloxane-divinylbenzene (PDMS/DVB) fibers (length = 1 cm), supplied by Supelco (Bellefonte, PA, USA), were employed in this study. Fiber was conditioned before use by inserting into the GC injector port at 250 °C during 30 min, following the manufacturer recommendations. A magnetic stirrer equipped with electronic contact thermometer (IKA^®^-Werke GmbH & Co. KG, Germany) was used to favor the volatilization of aroma-related compounds into the headspace.

The headspace solid phase micro-extraction (HS-SPME) process was carried out by introducing 10 g of fruit puree into a 20 mL glass vial, together with 1 g of sodium chloride and 40 µL of the IS solution at 1000 mg L^−1^. The vial was crimped with a polytetrafluoroethylene (PTFE)-faced septum, then immersed into a water bath at 65 °C, and magnetically stirred during 30 min for temperature equilibration. Afterwards, headspace sampling was performed for 20 min at 65 °C, stirring the solution at 800 rpm. Finally, the fiber was withdrawn from the vial and the SPME device was transferred to the GC injection port, where thermal desorption of the analytes was carried out at 240 °C for 5 min.

### 2.4. Chromatographic Analysis

The analyses were carried out on a gas chromatograph equipped with a flame ionization detector (GC-FID) from Agilent Technologies (Palo Alto, CA, USA), following a modification of the method described by Olbricht et al. [9]. The volatile compounds were separated on a CP-Wax 52 CB capillary column (30 m × 0.25 mm i.d., 0.25 µm film thickness) from Varian (Walnut CreeK, CA, USA), using helium as the carrier gas at a constant flow rate of 1 mL min^−1^. The injection port was equipped with a 0.75 mm i.d. splitless glass liner (Supelco, Bellefonte, PA, USA) and operated in the splitless mode at 240 °C. The oven temperature was initially maintained at 40 °C for 5 min, raised to 200 °C at 5 °C min^−1^ and held for 5 min, and then raised to 250 °C at 5 °C min^−1^ and held at this temperature for 25 min. The temperature of the FID was set at 250 °C. Under these experimental chromatographic conditions, 24 volatile compounds (+IS) were separated. The identification of volatile compounds was accomplished using an in-house database built by injecting standard compounds, dissolved in solvent and in matrix, under the same chromatographic conditions. Additional experiments were performed on a gas chromatograph (Varian CP 3800) coupled to mass spectrometry (Varian Saturn 2200) to increase the identification reliability using the NIST Mass Spectral Library. Quantification was performed in reference to the IS (mg of 2-octanol equivalents per kg of fresh fruit weight).

### 2.5. Experimental Design

The working variables for the HS-SPME procedure, namely equilibration time, time and temperature for analyte adsorption into the fiber, time and temperature for analyte desorption, as well as weights of sample and background electrolyte (i.e., sodium chloride), were optimized by using an experimental design methodology. To this end, a fractional factorial design (2^(7−3)^) with three center points was applied to evaluate the main effects of the SPME variables on the recovery of volatile compounds from strawberry samples. Study ranges for these variables were as follows: equilibration time, 5–30 min; extraction time, 20–30 min; extraction temperature, 25–65 °C; desorption time, 1–5 min; desorption temperature, 240–280 °C; weight of sample, 2.5–10 g; weight of background electrolyte, 10–25%. All the extraction tests were performed using a headspace volume in the range 30–50% of the total vial volume, and keeping constant the height of the fiber inside the vial, to ensure reproducibility and extraction efficacy, according to previous literature [26]. In total, 19 trials were conducted in random order using a pooled strawberry sample. To study the effects of the factors on the response variables (i.e., number of peaks and peak areas), a Pareto diagram was used. In this representation, the length of each bar is proportional to the standardized effect (i.e., estimated effect divided by its standard error), and the vertical line can be used to judge which effects are statistically significant at 95% confidence. Subsequently, a response surface methodology (RSM) based on central composite design (CCD) was developed to optimize those extraction factors that elicited significant effects on dependent variables [27]. CCD consisted of a two-level factorial design, a star design in which experimental points are at a distance α from its center, and the central point. Each factor was studied at five levels (−α, −1, 0, +1, +α). The relationship between dependent and independent variables was fitted by a second-order polynomial model. The validation of the quadratic model obtained by RSM was accomplished by analysis of variance (ANOVA).

### 2.6. Statistical Analysis

To investigate differences in the volatile profile among strawberry cultivars, the dataset was first submitted to analysis of variance (ANOVA, *p* < 0.05) with the Fisher’s Least Significant Difference (LSD) post hoc test. Complementarily, various pattern recognition techniques were applied for the preliminary exploration of data and the characterization of varieties (principal component analysis, PCA), and to discriminate between different samples (linear discriminant analysis, LDA). PCA is one of the most powerful and common techniques for reducing the dimensionality of large datasets without the loss of information. New variables obtained by a linear combination of the original ones are calculated in such a way to keep most of the information present in the original dataset in the lower number of new variables, named principal components (PCs). The eigenvalues generated during PCA give an indication of the amount of information carried by each component. To select the optimal number of components, one of the most used criteria is to retain only factors with eigenvalues higher than 1 (Kaiser criterion). The Bartlett’s sphericity test was used as a measure of the sampling adequacy for evaluating the suitability of performing a PCA in our dataset, which is particularly useful for small sample sizes. On the other hand, linear discriminant analysis (LDA) is a supervised classification tool based on the generation of a number of orthogonal linear discriminant functions equal to the number of categories minus one. The discriminant power of each variable is evaluated by measuring the value of the Wilks’ lambda parameter for the overall model after removing the selected variable. Then, a forward stepwise algorithm is used to select the variables to be included in the final model. According to this algorithm, the F value is used as a criterion for inclusion or removal of variables in the model. Thus, Wilks’ lambda and F values were used in the present work to check the significance of each predictor in the LDA accomplished. Prior to conducting multivariate statistical analyses, data were subjected to logarithmic transformation and Pareto scaling, and data normality was checked by inspecting probability plots. All statistical analyses were conducted on Statistica 7.0 software (StatSoft Inc., Tulsa, OK, USA).

## 3. Results and Discussion

### 3.1. HS-SPME Optimization and Validation

**Figure 1:**
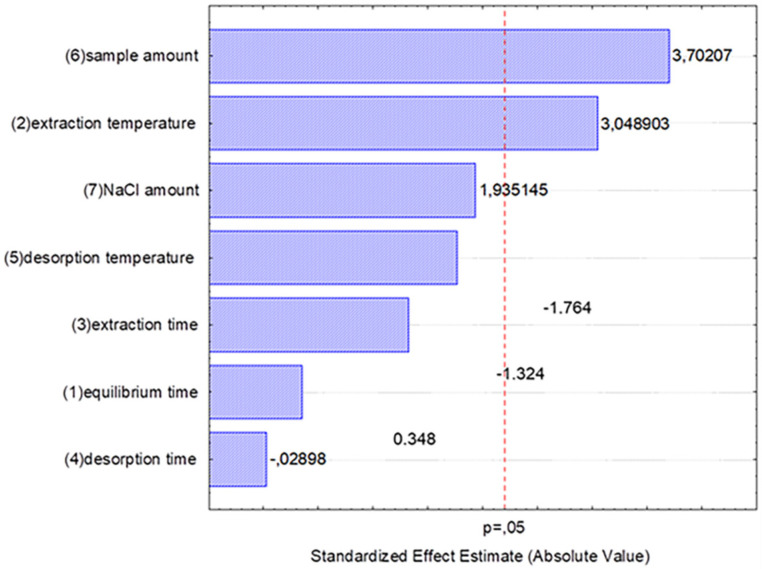
Pareto diagram showing the effect of factors investigated on the extraction efficiency.

Extraction efficiency in SPME not only depends on the polarity and thickness of the stationary phase, but is also affected by other variables such as agitation, time, temperature, or the addition of salts to the sample [28]. As a first step, we compared the performance of different fiber coatings for the extraction of volatile compounds from strawberry: polydimethylsiloxane (PDMS), polyacrylate (PA), polydimethylsiloxane/divinylbenzene (PDMS/DVB), carboxen/polydimethylsiloxane (CAR/PDMS), and divinylbenzene/carboxen/polydimethylsiloxane (DVB/CAR/PDMS). For this purpose, generic extraction conditions were applied based on our previous experience and literature: equilibration time, 15; extraction time, 30 min; extraction temperature, 50 °C; weight of sample, 10 g; weight of background electrolyte, 10%. The extraction efficiency was evaluated in terms of the total number of compounds extracted and their peak areas. The results evidence that, although different fibers were best suited for the extraction of certain compounds, the best overall extraction efficiency was obtained by using DVB/PDMS coatings, in line with previous studies [29,30,31]. Furthermore, we also checked that the HS volume did not have a significant influence on the extraction process if maintained in the range 30–50% of the total volume, thus providing suitable reproducibility and extraction recovery. To investigate the effect of other variables in the adsorption/desorption process, a two-level screening fractional factorial experimental design (2^(7−3)^) with three replicates in the center point was carried out. This screening experimental design evidenced a significant positive influence of sample weight and adsorption temperature on the extraction efficacy, which reached a plateau at the higher experimental conditions tested in this study. Accordingly, these parameters were subsequently optimized by using a central composite experimental design (CCD, with α = 1.414). To this end, sample weight and extraction temperature were tested in the ranges 6.5–12.5 g and 30–80 °C, respectively. All the other variables were kept at levels providing the best responses according to the Pareto diagram (Figure 1): equilibration time, 30 min; extraction time, 20 min; desorption time, 5 min; desorption temperature, 240 °C; weight of salt, 10%.

**Figure 2:**
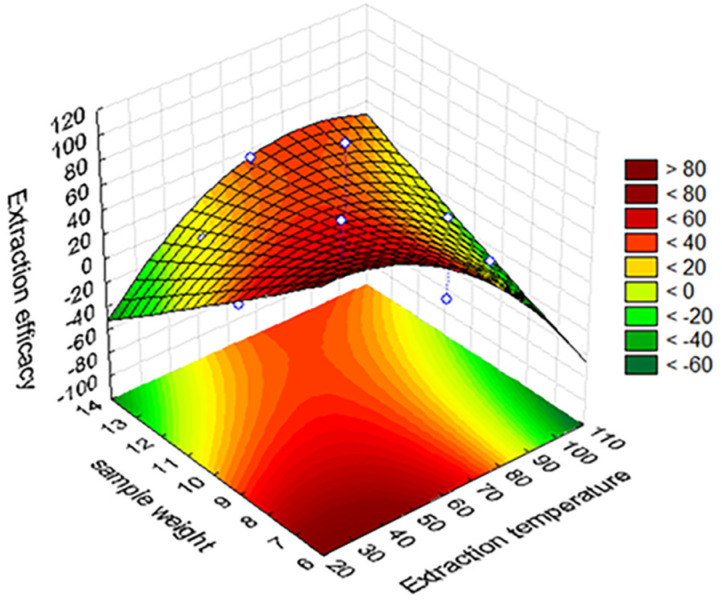
Response surface plots showing the effect of temperature and sample weight on the extraction efficiency according to the central composite design (CCD).

By applying the response surface methodology, the estimated optimum values for extraction temperature and sample weight were 65.4 °C and 11.4 g, respectively (Figure 2). The adequacy of the model was evaluated by using the coefficient of determination (R^2^), and assessing the regression fitness. The model showed statistical valid fitness (*p* < 0.05 for the regression F test) and non-significant lack of fit (*p* > 0.05). In addition, the coefficient of determination was estimated as 0.419.

To assess the precision of the extraction method previously optimized, nine independent extractions were carried out from the same batch of fruit puree at the optimum conditions. The extracts were prepared in three non-consecutive days, and replicated three times within each day. Repeatability was evaluated as the relative standard deviation of the ANOVA within-condition variance (S^2^_W_) considering the different days as the variation source. On the other hand, intermediate precision was evaluated from between-condition variance (S^2^_B_) and within-condition variance (S^2^_W_): S^2^_R_ = S^2^_W_ + S^2^_B_ [32]. The results evidence satisfactory repeatability, in the range 3.2% (ethyl butanoate) to 13.5% (hexanal), and intermediate precision, in the range 6.3% (geraniol) to 22.1% (hexanal). The limits of detection (LODs) and limits of quantification (LOQs) were calculated as the lowest concentration giving an average signal-to-noise (S/N) ratio above 3 and 10, respectively, by evaluating the background noise in blank samples. LOD values ranged from 0.42 µg mL^−1^ (for geraniol) to 3.53 µg mL^−1^ (for cis-nerolidol), whereas LOQs ranged from 1.41 µg mL^−1^ (for geraniol) to 11.78 µg mL^−1^ (for cis-nerolidol), expressed as IS equivalents. The analysis of standard solutions and spiked strawberry samples demonstrated the absence of significant degradation processes during the extraction (i.e., the signal intensity for standards dissolved in solvent that were analyzed without performing HS-SPME was comparable with that obtained after the extraction of the same standard solutions and spiked strawberry samples).

### 3.2. Characterization of the Strawberry Volatile Profile

**Figure 3:**
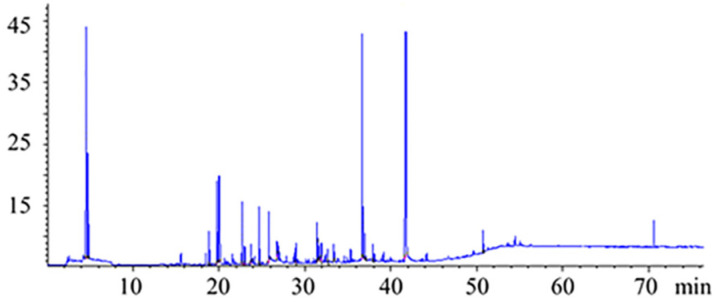
Typical chromatogram obtained by headspace solid phase micro-extraction coupled with gas chromatography (HS-SPME-GC) analysis of a Camarosa strawberry sample.

The HS-SPME-GC-FID methodology previously optimized was applied to characterize the volatile composition of three strawberry varieties grown in soilless system (Figure 3). In total, 24 compounds were identified in the samples under study, including esters, aldehydes, alcohols, acids, lactones, terpenes and furanones.

The concentrations of these volatile compounds in the three strawberry cultivars investigated are listed in Table 1. Esters were the most represented chemical class, with seven different compounds detected in, at least, two of the investigated cultivars. This agrees with previous works reporting that methyl and ethyl esters of butanoic and hexanoic acids are major volatiles in strawberry, responsible for its fruity and floral aroma [1,6,14,33]. According to the literature, terpenes (e.g., cis-nerolidol) and furans (e.g., furaneol, mesifurane) are also important contributors to the typical strawberry-like aroma [1,8,34], which were also detected in the present study at high concentrations.

The results evidence that the volatile profile is significantly different between strawberry varieties, both qualitatively and quantitatively, in line with previous studies reported in literature (Table 2). Esters were the most abundant chemical class in the Camarosa cultivar (22%), followed by terpenes (19.1%) and acids (12.2%). On the other hand, Candonga and Festival varieties were richer in terpenes (31.6 and 34.8%, respectively), followed by esters (24.7 and 26.4%, respectively). Specifically, methyl butyrate, hexyl hexanoate, linalool, geraniol and furaneol were the most abundant volatile compounds, whereas methyl anthranilate was not detected in any of the analyzed samples. As shown in Table 1, the Camarosa variety was characterized by significantly lower levels of most of the volatiles investigated, including esters (e.g., ethyl butanoate, ethyl hexanoate, hexyl hexanoate), terpenes (e.g., linalool, geraniol), and furanones (e.g., furaneol). Ethyl hexanoate was not detected in any of the samples analyzed for this variety, and mesifuran was only found in one of the samples analyzed, in line with the results obtained by Ubeda et al. for some strawberry varieties (Fuentepina, Camarosa, Candonga, Sabrina) [7]. Conversely, cis-nerolidol was majorly detected in Camarosa strawberries, at levels similar to those previously reported [35]. On the contrary, Festival samples showed the highest total content of esters, terpenes and furanones (Table 1), although some specific compounds were not detected (e.g., hexanoic acid, benzyl alcohol, benzaldehyde, cis-3-hexenyl acetate). It should be noted that furaneol concentration in Festival cultivar was almost five times higher than in the Camarosa variety and almost double that in Candonga, being this volatile molecule a key aroma compound in strawberry according to the literature [1,7,8,33,34]. Finally, the volatile levels in Candonga strawberries were, in general, between those found for the other two cultivars investigated. All these results therefore suggest that Festival strawberries potentially have a more aromatic profile than Candonga and, especially, Camarosa ones, with higher concentrations for most of the differential volatile compounds listed in Table 1. However, these results should be validated by comparing these concentrations with sensory thresholds.

### 3.3. Chemometrics Approaches to Identify Chemical Descriptors of Strawberry Cultivar

**Figure 4:**
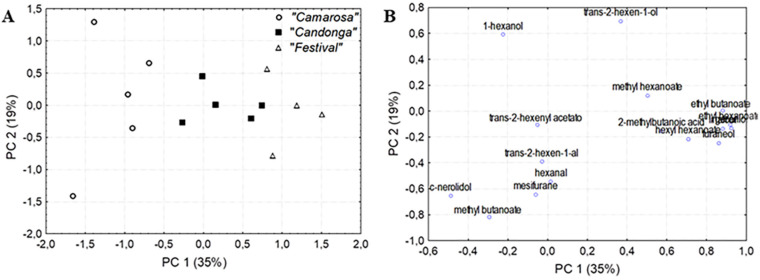
Principal component analysis (PCA) scores plot showing the distribution of samples in the space defined by the two first principal components (**A**); and a PCA loadings plot showing the contribution of each variable to the two first principal components (**B**).

To investigate the potential of volatile metabolites as chemical descriptors to discriminate between strawberry cultivars grown in soilless systems, various complementary chemometrics approaches were employed in the present study. Principal component analysis (PCA) was first applied for a rapid exploration and visualization of the data trends. The PCA model obtained, with three principal components (PC), was able to explain up to 69.4% of the total variance. As shown in the corresponding scores plot (Figure 4A), the three strawberry varieties were slightly separated along the first principal component, but significant intra-cultivar variability was also observed. The PC1 was positively associated with levels of ethyl butanoate, ethyl hexanoate, hexyl hexanoate, linalool, geraniol and furaneol (Figure 4B). That is, the volatile content was in general higher in Festival samples, located in the right side of the PCA plot with positive loadings, in line with results presented in Table 1. On the other hand, the main contributors to PC2 were trans-2-hexen-1-ol, 1-hexanol, methyl butanoate, mesifurane and cis-nerolidol, thereby being responsible for the variability detected within Camarosa strawberries and, consequently, their wide spread on PC2 (Figure 4A).

In a second step, linear discriminant analysis (LDA) was applied to build classification models for the predefined groups and to select the variables with higher discriminant power. For this purpose, the dataset was randomly divided into two groups, i.e., training and validation sets, accounting for 80% and 20% of the total number of samples, respectively. The training set was used to build the model, whereas the validation set enabled the testing of its performance. To validate the recalling rate (effectiveness of classification in the training set) and the prediction ability (effectiveness of classification in the validation set) of the LDA model, both training and validation sets were repeated at least ten times with different constitutions. The average percentage of correctly classified cases in the recalling and prediction tests obtained from these 10 runs was used as a measure of the method performance. Using this method, two significant discriminant functions (roots) were obtained by stepwise LDA (Wilks’ lambda *p*-values 0.0000 and 0.0001, respectively), which explained 68% and 32% of the total variability of the data, respectively. As shown in Table 3, the most discriminant variables were geraniol, hexyl hexanoate, and trans-2-hexen-1-al.

**Figure 5:**
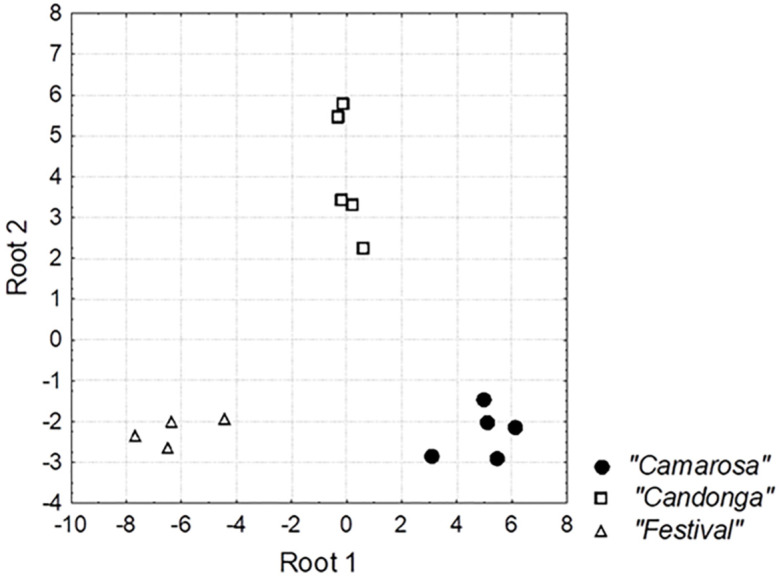
Linear discriminant analysis (LDA) scatterplot showing the distribution of samples in the space defined by the two discriminant functions.

This LDA model yielded satisfactory classification (92.18%) and prediction (75.68%) rates, thereby allowing a clear discrimination among the study groups, as illustrated in Figure 5. The first discriminant function enabled the separation of the three varieties, mainly Festival and Camarosa cultivars with negative and positive loadings for root 1, respectively. The primary variables responsible for the discrimination of Festival strawberries with respect to the other two varieties were geraniol and hexyl hexanoate, with negative loadings in root 1 (Table 3). On the other hand, Candonga samples were orthogonally separated in the second root. This second discriminant function was positively associated with geraniol and negatively with trans-2-hexen-1-al, which could therefore serve as chemical descriptors for the Candonga cultivar.

## 4. Conclusions

In this study, we aimed to investigate the varietal differences in the volatile profile of strawberries cultivated in soilless systems. To this end, we have developed, in the present study, a method based on headspace solid phase micro-extraction coupled with gas chromatography (HS-SPME-GC-FID) for characterizing the volatile fraction of strawberry. This methodology enabled the detection of 24 aroma-related compounds in strawberry fruits grown in a soilless system. Furthermore, we observed that the levels of these metabolites were highly influenced by genotypic factors, with the Festival cultivar having higher concentrations for most of the differential volatile compounds here assayed. The application of complementary chemometrics approaches suggested that some of these molecules, namely geraniol and hexyl hexanoate, might be employed as chemical descriptors to discriminate between different strawberry cultivars.

## Figures and Tables

**Table 1:** Volatile composition of three strawberry varieties grown in a soilless system (expressed as the mean ± standard deviation of 2-octanol equivalents, µg kg^−1^) and *p*-values obtained by ANOVA (N = 5 per study group).

	Camarosa	Candonga	Festival	*p*-Value
Esters				
methyl butanoate	142.21 ± 14.65 ^a^	112.72 ± 10.59 ^b^	99.63 ± 7.92 ^b^	0.0002
ethyl butanoate	14.37 ± 1.62 ^a^	28.01 ± 2.31 ^a^	48.06 ± 3.14 ^b^	0.01
methyl hexanoate	24.51 ± 2.75 ^a^	62.31 ± 3.20 ^b^	57.64 ± 4.04 ^b^	0.0001
ethyl hexanoate	ND	19.26 ± 3.31 ^a^	28.48 ± 4.31 ^b^	0.0006
hexyl hexanoate	95.45 ± 16.59 ^a^	150.47 ± 24.00 ^a^	303.79 ± 94.39 ^b^	0.0063
cis-3-hexenyl acetate	40.71 ± 9.10	42.52 ± 8.37	ND	NS
trans-2-hexenyl acetate	24.77 ± 1.90	33.66 ± 3.39	26.44 ± 2.33	NS
Aldehydes				
hexanal	26.41 ± 9.51	18.24 ± 4.45	26.43 ± 6.54	NS
trans-2-hexen-1-al	19.75 ± 2.24 ^ab^	6.78 ± 0.92 ^a^	21.96 ± 4.44 ^b^	0.0055
benzaldehyde	29.21 ± 6.53	21.68 ± 4.85	ND	NS
Alcohols				
1-hexanol	38.25 ± 2.10	21.83 ± 4.07	34.77 ± 2.19	NS
trans-2-hexen-1-ol	30.94 ± 12.24	31.96 ± 15.13	50.76 ± 18.35	NS
benzyl alcohol	12.68 ± 2.09	6.60 ± 1.47	ND	NS
Terpenes				
linalool	75.08 ± 14.63 ^a^	135.00 ± 21.74 ^b^	188.74 ± 32.02 ^b^	0.0053
geraniol	151.63 ± 29.96 ^a^	426.43 ± 35.72 ^b^	537.73 ± 122.01 ^b^	0.0001
cis-nerolidol	51.77 ± 7.60 ^a^	5.91 ± 1.32 ^b^	12.69 ± 2.46 ^b^	0.0001
nerol	7.59 ± 1.70 ^a^	7.73 ± 1.73 ^a^	4.33 ± 0.86 ^b^	0.0053
Furanones				
mesifurane	27.83 ± 6.22	33.84 ± 5.08	39.80 ± 8.12	NS
furaneol	96.56 ± 24.29 ^a^	210.38 ± 47.73 ^b^	456.97 ± 117.81 ^c^	0.00001
Acids				
2-methylbutanoic acid	30.71 ± 5.67 ^a^	64.72 ± 16.26 ^b^	46.23 ± 6.14 ^a^	0.0017
3-methylbutanoic acid	109.32 ± 22.48 ^a^	89.26 ± 15.81 ^a^	4.63 ± 0.92 ^b^	0.0001
hexanoic acid	43.01 ± 9.60	29.04 ± 6.42	ND	NS
Lactones				
γ-nonalactone	9.89 ± 2.21	11.69 ± 2.61	ND	NS
Δ-decalactone	4.83 ± 1.08	20.36 ± 4.55	ND	NS

Superscript letters within each row indicate significant differences between groups marked with different letters, according to the post-hoc LSD test (*p* < 0.05). ND: not detected, NS: not significant (*p* > 0.05).

**Table 2:** Summary of previous works investigating the influence of the variety in the strawberry volatile profile.

Varieties Investigated	Findings	Reference
35	Thirty-one volatile compounds correlated to strawberry flavor intensity, particularly esters, terpenes and furans.	[1]
4	Key odorants identified were furaneol, γ-decalactone, ethyl butanoate, ethyl hexanoate, ethyl 3-methylbutanoate, diacetyl and hexanoic acid. The aroma of Fuentepina and Candonga varieties presented mainly green notes, whereas the aromatic notes in Camarosa and Sabrina varieties were mainly sweet.	[7]
12	The most abundant volatile sulfur compounds in strawberry are methanethiol, dimethyl sulfide, dimethyl disulfide and dimethyl trisulfide, being methanethiol the predominant aromatic compound. Festival and Florida Radiance presented higher thioester concentrations., whereas Dover, Rosa Linda and Florida Belle were characterized by relatively high sulfide and low thioester concentrations.	[10]
9	Esters, such as methyl butanoate, pentyl acetate and methyl hexanoate, characterized the aroma of ripe strawberries, and allow discriminating between cultivars.	[11]
16	Great diversity of the volatile patterns in F. vesca accessions in comparison with F. × ananassa cultivars.	[33]
5	The content of volatiles varied depending on the cultivars, but in general ethyl butanoate, mesifurane, ethyl hexanoate, ethyl 3-methylbutanoate, hexyl acetate and γ -dodecalactone had the highest odor activity values.	[35]

**Table 3:** Summary of the statistical performance for the linear discriminant analysis (LDA) model.

		Root 1	Root 2
Canonical correlation		0.9808	0.9597
Eigenvalue		25.33009	11.66195
Cum. Prop		0.68474	1.00000
variables	*F*-value ^a^	correlation of variables with roots	
geraniol	23.26316	−0.450750	0.217442
hexyl hexanoate	18.57001	−0.299080	−0.093153
trans-2-hexen-1-al	6.17330	−0.021863	−0.235022

^a^ significant at *p* < 0.001.
